# Experimental Investigation of Wavy-Lap Bonds with Natural Cotton Fabric Reinforcement under Cyclic Loading

**DOI:** 10.3390/polym13172872

**Published:** 2021-08-26

**Authors:** Viktor Kolář, Miroslav Müller, Martin Tichý, Rajesh Kumar Mishra, Petr Hrabě, Kristýna Hanušová, Monika Hromasová

**Affiliations:** 1 Department of Material Science and Manufacturing Technology, Faculty of Engineering, Czech University of Life Sciences Prague, Kamycka 129, Suchdol, 16500 Prague, Czech Republic; vkolar@tf.czu.cz (V.K.); muller@tf.czu.cz (M.M.); martintichy@tf.czu.cz (M.T.); hrabe@tf.czu.cz (P.H.); hanusovakristyna@tf.czu.cz (K.H.); 2 Department of Electrical Engineering and Automation, Faculty of Engineering, Czech University of Life Sciences Prague, Kamycka 129, Suchdol, 16500 Prague, Czech Republic; hromasova@tf.czu.cz

**Keywords:** quasi-static test, cyclic fatigue, wavy-lap bond, natural cotton fabric, polymer composite, mechanical properties, service life, safety, SEM

## Abstract

This study is focused on the mechanical properties and service life (safety) evaluation of hybrid adhesive bonds with shaped overlapping geometry (wavy-lap) and 100% natural cotton fabric used as reinforcement under cyclic loading using various intensities. Cyclic loading were implemented between 5–50% (267–2674 N) and 5–70% (267–3743 N) from the maximum strength (5347 N) measured by static tensile test. The adhesive bonds were loaded by 1000 cycles. The test results demonstrated a positive influence of the used reinforcement on the mechanical properties, especially during the cyclic loading. The adhesive bonds Tera-Flat withstood the cyclic load intensity from 5–70% (267–3743 N). The shaped overlapping geometry (wavy-lap bond) did not have any positive influence on the mechanical performance, and only the composite adhesive bonds Erik-WH1 and Tera-WH1 withstood the complete 1000 cycles with cyclic loading values between 5–50% (267–2674 N). The SEM analysis results demonstrated a positive influence on the fabric surface by treatment with 10% NaOH aqueous solution. The unwanted compounds (lignin) were removed. Furthermore, a good wettability has been demonstrated by the bonded matrix material. The SEM analysis also demonstrated micro-cracks formation, with subsequent delamination of the matrix/reinforcement interface caused by cyclic loading. The experimental research was conducted for the analysis of hybrid adhesive bonds using curved/wavy overlapping during both static and cyclic loading.

## 1. Introduction

Adhesive bonding technology represents one of the most promising methods of material bonding. This technology finds its application in automotive, aviation, and electrotechnical industries [1,2]. The dynamic development of adhesive bonding technology is demonstrated by the wider possibilities offered by this process as compared to the conventional technologies of bonding materials (welding, soldering, etc.). Significant advantages are observed as compared to conventional technologies in a wide spectrum of bonding materials, along with lower component costs and lower labor requirements [3]. Adhesive technology could also fulfill supporting roles, such as sealing, clamping, and securing [4,5]. Currently, there are plenty of research opportunities dealing with adhesive bonding. Their aim is to improve the efficiency of using such material under loading conditions. The majority of research deals with the static strength of adhesive bonds [6,7,8]. The mechanical properties of adhesive bonds could be influenced by physical and chemical factors (wettability, adhesion and cohesion, aging, and environmental degradation) [4,9,10], technological factors (roughness and structure of bonded surface and filler material) [11,12,13], and constructional factors (overlapping length and geometry and type of applied load) [14,15,16,17,18]. The resulting performance of the adhesive bond is governed by the synergy of these factors, i.e., the effect of their mutual interaction [19].

Shaped overlapping geometry is one of the factors that could positively influence the adhesive bond strength and the internal stress [20,21]. Another reason for using shaped overlapping geometry is to address a more complex constructional requirement of the adhesive systems when non-flat-lap bonds are used. Many researchers have dealt with shaped overlapping geometry. Zeng and Sun [22] came up with a solution of wavy-lap bonds and detected an increase in shear strength as compared to flat-lap bonds during a static test. Ávila and Bueno [23] conducted a similar experiment and detected an increase of 41% in shear strength under the static test. Müller [24] tested the influence of various adhesive types on the strength of wavy-lap bonds. A number of researchers are devoted to modification of various types of wavy-lap bonds. Jaiswal et al. [25] tested adhesive bonds with teeth of different depth created on the lap surface to increase the static tensile strength. Haghpanah et al. [26] tested adhesive bonds with different adherend geometry using positive and negative teeth. Razavi et al. [27] dealt with sinusoidal geometry of adherend lapping in their research.

The wettability of natural fiber reinforcement is a significant factor influencing its bonding properties. Deteriorated wettability (adhesion) of natural-fiber-based reinforcements, which usually decreases the shear strength of adhesive bonds, leads to significant disadvantages for their use in the polymer composites [28,29,30,31,32]. Deteriorated wettability of such natural fiber surfaces could be minimized by chemical treatment in aqueous solution of NaOH, plasma treatment of its surface, or by other methods. [33]. Alkali treatment with NaOH solution improves the surface structure of the reinforcement. The improvement is caused by removal of unwanted layers, e.g., lignin, oils, and fats, from the reinforcement fiber surface [34,35,36]. The surface treatment leads to improvement of interaction at the interphase boundary, i.e., on the interface of the natural reinforcement and matrix [37]. It leads to improvement of mechanical properties, especially the shear strength of adhesive bonds [19,35,38].

During application, the bonded materials are loaded, not only under static condition, but also by cyclic loading. A number of studies have dealt with cyclic loading of different intensities in the field of fiber–polymer composites [39,40]. With adhesive bonds, it cannot be expected that quality will be preserved throughout their service life. Operating conditions usually include the action of the cyclic loading, i.e., cyclic fatigue. Cyclic fatigue is characterized by propagation of cracks inside of the adherend and subsequent permanent damage to the adhesive bonds [41]. The process itself leads to relatively lower values of cyclic loading due to delamination between adherend and bonded material, which negatively influences the service life of the adhesive bonds [42]. The strength and fatigue service life of adhesive bonds are even lower at smaller numbers of repeating cycles. The tests of cyclic loading are essential for practical application of adhesive bonds [42,43,44]. The researchers demonstrated that the hybrid composite layer of adhesive bonding can positively influence the mechanical properties and extend their service life under cyclic loading [19,35,45].

The experimental research was mainly focused on the hybrid adhesive bonds with shaped overlapping geometry (wavy-lap) and 100% natural cotton fabric as reinforcement. Adhesive bonds were exposed to cyclic loading of various intensities, and the results of mechanical properties and service life (safety) were evaluated. Cyclic loading (cyclic fatigue) represents a common cause of failure in adhesive bonds due to delamination of reinforcement and the matrix. Based on previous research to achieve optimum results for mechanical properties and service life during cyclic loading, the bonding materials and procedure were chosen. That included selection of 100% cotton fabric as reinforcement [19]. The previous studies have focused on flat geometries, while some of the real applications are in the form of curved (wavy) shapes.

The aim of this study’s research was to evaluate the influence of the shaped adherend geometry (wavy-lap) and reinforcing natural cotton fabric with modified surface. 10% aqueous solution of NaOH was used for pretreatment of the cotton fabrics. Mechanical properties (tensile strength, deformation-strain, modulus of elasticity) and related service life and safety of the hybrid adhesive bonds with composite layer of adhesive was evaluated by cyclic loading of various intensities. Selected mechanical parameters provide an overview of the behavior of adhesive bonds and cyclic loading to approximate realistic loading conditions and their subsequent application.

## 2. Materials and Methods

### 2.1. Materials

#### 2.1.1. Bonded Material (Adherend)

Structural carbon steel S235J0 (Ferona a.s., Prague, Czech Republic) with dimensions 1 mm thickness, 100 ± 0.25 mm length, and 25 ± 0.25 mm width was used as an adherend. The adherend dimensions were established by the ČSN EN 1465 standard [46]. Basic mechanical properties and indicative chemical composition are listed in Table 1 and Table 2.

**Figure 1:**
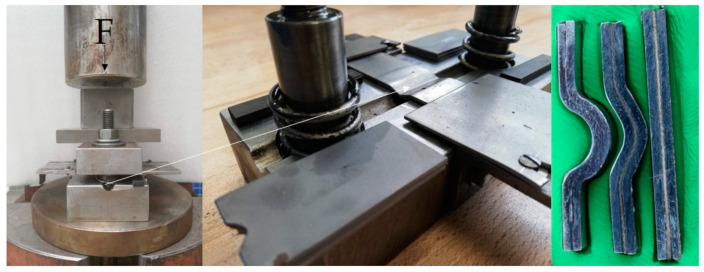
Pressing form for the shaping of adherends.

**Figure 2:**
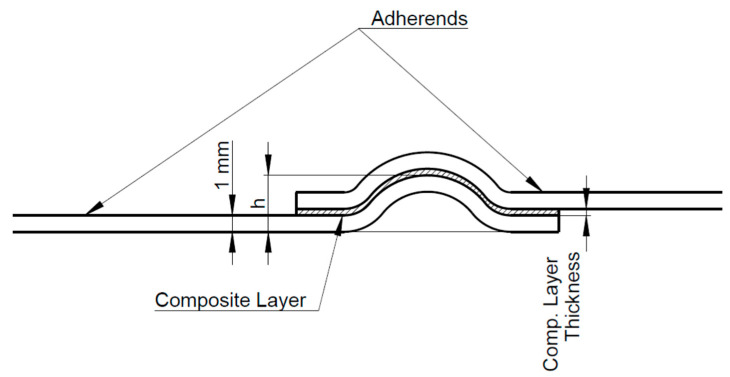
The types of shaped adherends and the measurement principle for height *(h)* of a wave.

The shaping of the adherends (height *h* of wavy-lap bonds) was achieved by using a pressing form (Figure 1). The adherends were placed in a form, and by using 850 N (F) force, the shaped geometry with different wave heights h_1_ = 2.43 ± 0.10 mm (marked as WH1) and h_2_ = 4.82 ± 0.13 mm (marked as WH2) was obtained. The types of adherends and principle of measurement for the height (*h)* of a wave are shown in Figure 2.

The surface of the adherends was mechanically treated in a blasting cabin using abrasive Garnet MESH 80 and then chemically treated in an acetone bath just before bonding. These methods for surface treatments were proven as optimal in terms of mechanical properties of adhesive bonds by several studies [45,48]. The roughness of adherends’ surfaces were measured using profilometer Mitutoyo Surftest 301 (Mitutoyo Europe GmbH, Neuss, Germany). Value *Ra* = 3.65 ± 0.12 µm and *Rz* = 11.19 ± 0.37 µm.

#### 2.1.2. Matrix and Reinforcement

2 types of 100% natural cotton fabric were used as reinforcement. Their characteristics are listed in Table 3.

The surface of natural cotton fabric was alkali-treated before application of the adhesive layer. Treating the surface leads to improvement of wettability and thus improves the performance of the bond, mainly its strength [33,49,50]. The following steps were used for the surface treatment:

1. Soaking the fabrics in hot water (100 °C) for removal of starch;
2. Rinsing with cold water for removal of residual impurity;
3. Soaking the fabrics in 10% NaOH solution for 30 min. Distilled water was used to create the solution;
4. Repeated washing of the alkali-treated fabrics with cold water;
5. Drying the fabric in a laboratory oven at 105 °C temperature for 24 h [51].

Structural two-component epoxide resin CHS-Epoxy 324 (Epoxy 1200) (Havel Composites CZ s.r.o., Svésedlice, Czech Republic) with P11 hardener (Havel Composites CZ s.r.o., Svésedlice, Czech Republic) was used as a matrix (in weight ratio 100:7 according to the manufacturer’s recommendation). According to the manufacturer, resin is suitable for metal bonding [52].

#### 2.1.3. Preparation of Adhesive Bonds

The research was based on modified norm ČSN EN 1465. The norm ČSN EN 1465 establishes the lapping length to be 12.5 ± 0.25 mm. The length of the lapping was based on shaped adherend geometry and was identical for all of the adhesive bond types (29 ± 1.31 mm) so that the results could be compared. The bonds were loaded with 750 g (7.4 N) weights and left to be hardened at 21 ± 2 °C laboratory temperature and 45 ± 7% relative air humidity for 24 h. The adhesive layer thickness was measured using Gwyddion software (version 2.49, David Nečas and Petr Klapetek, VUT Brno, Brno) from scanning electron microscope (SEM) images. Type, shape, and adhesive layer thickness of the hybrid adhesive bonds are listed in Table 4.

### 2.2. Methods

The testing of mechanical properties was realized on a universal testing machine LABTest 5.50 ST (LABORTECH s.r.o., Opava, Czech Republic) with measuring unit AST KAF 50 kN (LABORTECH s.r.o., Opava, Czech Republic) and evaluation software Test & Motion (version 4.5.0.15, LABORTECH s.r.o., Opava, Czech Republic) at 21 ± 1 °C laboratory temperature and 44 ± 4% relative air humidity. The testing of mechanical properties during cyclic loading, i.e., tensile strength and extension upon rupture, was based on setting the standard value obtained during static tensile test (ČSN EN 1465) consisting of 7 adhesive bonds marked as Resin-Flat with testing speed 0.6 mm × min^−1^. The testing speed during static test was chosen on the basis of the ČSN EN 1465 standard, which defines the test duration in the interval of 60 ± 2 s.

The maximum average load of 5347 ± 157 N (average value from 7 Resin-Flat adhesive bonds) was obtained. Cyclic loading (quasi-static test) consisted of 1000 cycles with testing speed 6 mm × min^−1^ within the limits of 5%, 50% and 70% of maximum load. The lower limit was 5% = 267 N and the upper limit was 50% and 70% from the maximum load, i.e., 50% = 2674 N and 70% = 3743 N. The testing speed during the cyclic test was chosen based on the characteristics of cyclic loading, which often results in sharp fluctuations in its intensity. For this reason, the test speed was higher than for static tests. The time delay between lower and upper limit was set for 0.5 s. When 1000 cycles were finished, a static tensile test automatically followed and ran until complete failure of adhesive bond with 0.6 mm × min^−1^ speed. Static test was only realized if 1000 cycles were finished. If they were not, the test was concluded. Every testing sequence consisted of 7 testing samples.

The analysis of variance was used to evaluate the executed experiments, i.e., ANOVA-F test in STATISTICA 12 (version 12, StatSoft CR, Prague, Czech Republic) program. The Resin-Flat was set as the reference. The statistical dependency of 0.05 limit (95% confidence interval) between average and each experiment variant was evaluated. The null hypothesis H_0_ presents a statistically insignificant difference between measured data (*p* > 0.05). Alternative hypothesis H_1_ rejects null hypothesis H_0_ and presents statistically significant difference between measured data (*p* < 0.05).

Hybrid layer of adhesive bonds was evaluated using scanning electron microscope MIRA 3 TESCAN GMX SE (Tescan Brno s.r.o., Brno, Czech Republic). The interaction at interphase boundary between reinforcement/matrix and adherend/composite layer was evaluated. Samples were coated with gold using Quorum Q150R ES (Tescan Brno s.r.o., Brno, Czech Republic) device for the microscopy.

## 3. Results and Discussion

Strength of adhesive bonds depends on many factors. An important factor is the overlapping length, i.e., the area that conveys adhesion stress. It is not possible to apply a random amount of adhesive layer during practical application. This restriction is due to the increasing weight, constructional limitations, and shape complexity of the final product. This study focuses on surface modification before adhesive bonding using forming, specifically, adherend forming using a specific angle. Eventually, a wavy profile forming on the surface helped the wetting of adhesive become more efficient [24,53,54,55,56,57,58]. The wavy geometrical shape of a bonded surface usually had a positive effect on the tensile strength of adhesive bonds [24,53,54,55,56,57,58]. However, the results did not demonstrate a significant influence of geometry of adhesive bonded surface by using two types of bonded material. A significant improvement was observed by using reinforcing cotton fabric in the hybrid adhesive layer. This was demonstrated by an increase in service life of the adhesive bond during low-cycle fatigue, an essential aspect for adhesive bond application.

**Figure 3:**
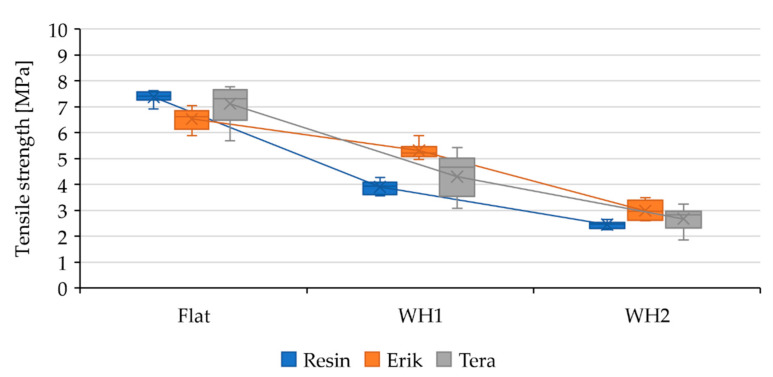
Evaluation of the tensile strength of adhesive bonds under static loading and their dependance on the bond shape.

**Figure 4:**
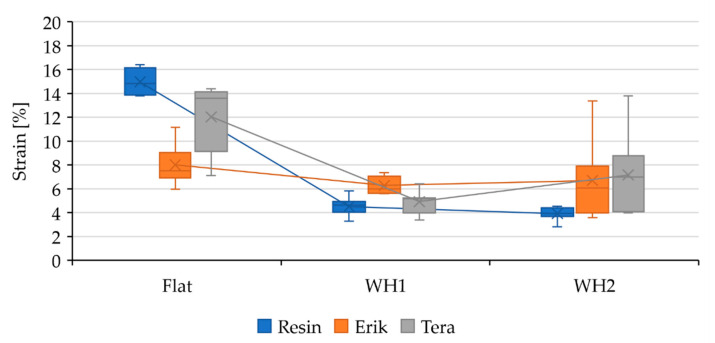
Evaluation of the strain in adhesive bonds under static loading and their dependance on the bond shape.

**Figure 5:**
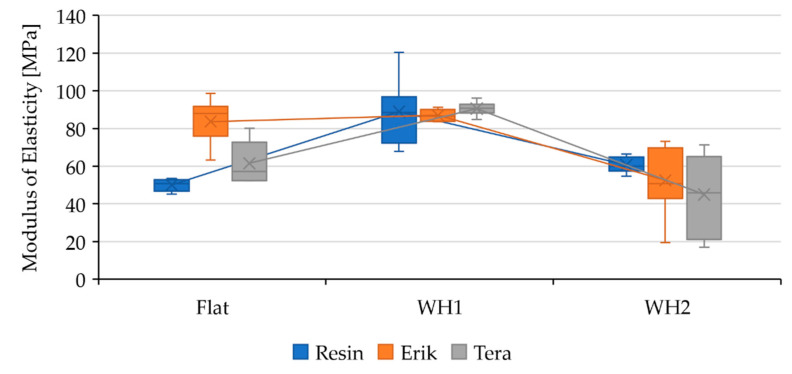
Evaluation of the modulus of elasticity of adhesive bonds under static loading and their dependance on the bond shape.

Adhesive bonds were initially evaluated by static tensile test. The mechanical properties of adhesive bonds (Resin, Erik, and Tera) and different lapping construction (Flat, WH1, and WH2) under static tests are listed in Table 5. The influence of the shape change along with the reinforcement fabrics on mechanical properties is described based on their dependency in Figure 3, Figure 4 and Figure 5 where the data are compared to the result of Resin-Flat bonds.

The static tensile test results demonstrated a quite severe deformation, 14.3 ± 1.88%, for the Resin-Flat adhesive bond, as shown in Table 5. The adhesive bond strength, however, was the highest, 7.38 ± 0.22 MPa, among all tested samples. The change of geometry from standard lapped bond Resin-Flat construction to shaped lapped bonds Resin-WH1 and Resin-WH2 did not have a positive influence on the tensile strength during static tests. As shown in Figure 3, the strength of Resin-WH1 decreased by 47% to 3.91 ± 0.23 MPa, and that of Resin-WH2 decreased by 67% to 2.45 ± 0.13 MPa.

The tensile strength of Erik-Flat decreased slightly by 10% to 6.53 ± 0.38 MPa compared to Resin-Flat. The strength in Erik-WH1 decreased by 28% to 5.31 ± 0.29 MPa. The drop in this case is not as big as by Resin-WH1. The strength in Erik-WH2 decreased by 60% to 2.99 ± 0.33 MPa. This drop was 7% lower in sample Resin-WH2. The results clearly demonstrate that the Erik fabric positively influenced the tensile strength in samples Erik-WH1 and WH2, as seen in Figure 3.

The Tera-Flat achieved 7.12 ± 0.74 MPa strength during static loading, which is 3% lower compared to Resin-Flat. It is, however, statistically insignificant. The strength in Tera-WH1 decreased by 42% to 4.30 ± 0.83 MPa. This drop was 5% lower than sample Resin-WH1. The strength in Tera-WH2 decreased by 64% to 2.67 ± 0.43 MPa, 3% lower than Resin-WH2. The results demonstrate that the Tera fabric slightly increased the tensile strength of Tera-WH1 and Tera-WH2, as seen in Figure 3.

The static tensile test results demonstrated quite severe deformation (strain), 14.3 ± 1.88%, in Resin-Flat, as seen in Figure 4 A severe deformation points to a rather low load-bearing capacity of the bond. Previous research also shows that an adhesive bond with such severe deformation cannot withstand cyclic loading [19]. This fact was proven by cyclic loading in 5–50% (267–2674 N) and 5–70% (267–3743 N) intervals, where the adhesive bond with pure resin did not withstand the load in any of the intervals. The fracture area showed adhesive-cohesive structure. The endurance of the resin bond was not influenced by WH1 and WH2 modification. In sample Resin-WH1, the deformation decreased to 4.51 ± 0.73%, as shown in Table 5. In Resin-WH2, the deformation again decreased to 3.92 ± 0.54%. Too-moderate deformation with moderate strength shows low endurance of the bond during cyclic loading [19]. That is why the adhesive joints did not withstand the cyclic loading.

Deformation in Erik-Flat positively decreased to 8 ± 1.59%. This drop defines an increase in the bond rigidity while maintaining strength and thus improved endurance during cyclic tests, as shown in Figure 3 and Figure 4. The deformation in Erik-WH1 positively decreased to 6.27 ± 0.65%. Even though the construction/geometry of the bond was changed, the rigidity was preserved, resulting into endurance of the bond during cyclic loading in a 5–50% interval. Erik-WH2 showed a higher deformation, 6.69 ± 3.12%, associated with a lower strength, as shown in Figure 3 and Figure 4. This demonstrates a lower endurance under cyclic loading.

Similar deformation occurred in the case of Tera-Flat. The observed deformation was 12.03 ± 2.70%, which is lower only by 2.3% (statistically insignificant, *p*-value 0.22). This small difference in deformation caused a sufficient increase in rigidity of the bond under cyclic loading, in both 5–50% and 5–70% intervals. Deformation in Tera-WH1 positively decreased to 4.92 ± 0.91%, maintaining optimal ratio between strength and deformation and thus the rigidity of adhesive bond, as shown in Figure 3 and Figure 4. Tera-WH2 showed 7.16 ± 3.14% deformation. That is a rather huge deformation associated with a lower strength. That demonstrates a low endurance during cycling loading.

Figure 5 shows the modulus of elasticity in the bonded samples. The Resin-Flat bond exhibited a modulus of 52.46 ± 6.65 MPa. The modulus in the Erik-Flat bond increased to 83.68 ± 10.87 MPa. In the case of the Tera-Flat bond, the modulus increased to 61.53 ± 10.43 MPa. The Erik-Flat and Tera-Flat samples showed higher modulus of elasticity and thus improved performance under cyclic loading. The Resin-WH1 bond showed a modulus of 88.96 ± 16.02 MPa, while the Erik-WH1 bond showed 85.18 ± 5.29 MPa and Tera-WH1 showed 87.61 ± 7.91 MPa. In the case of wavy-shaped bond WH1, there was an increase in the modulus of elasticity. The Resin-WH2 bond exhibited a lower modulus of 63.67 ± 8.14 MPa. Erik-WH2 bond showed a modulus of 52.48 ± 17.16 MPa, and Tera-WH2 showed 44.99 ± 19.14 MPa. Wavy-shaped bond WH2 exhibited a lower modulus compared to WH1, which would affect endurance and fatigue properties under cyclic loading.

The results of cyclic mechanical tests of adhesive bonds with reinforcing fabrics Tera and Eric with different bond shapes are listed in Table 6. The results of the static tests showed that Resin-Flat, WH1, and WH2 did not withstand any intensity of cyclic loading. Wave-shaped geometries of Resin-WH1 and WH2 did not affect mechanical properties during static test positively enough to be able to resist the cyclic loading. As a result, neither of the shaped adhesive bonds with pure resin performed well during the cyclic loading in 5–50% (267–2674 N) and 5–70% (267–3743 N) intervals.

**Figure 6:**
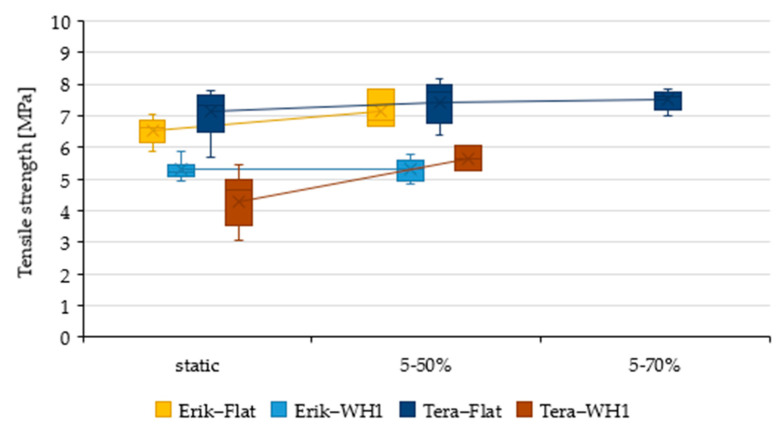
Evaluation of the tensile strength of the adhesive bonds under static loading and cyclic loading in the load intervals 5–50% and 5–70%.

**Figure 7:**
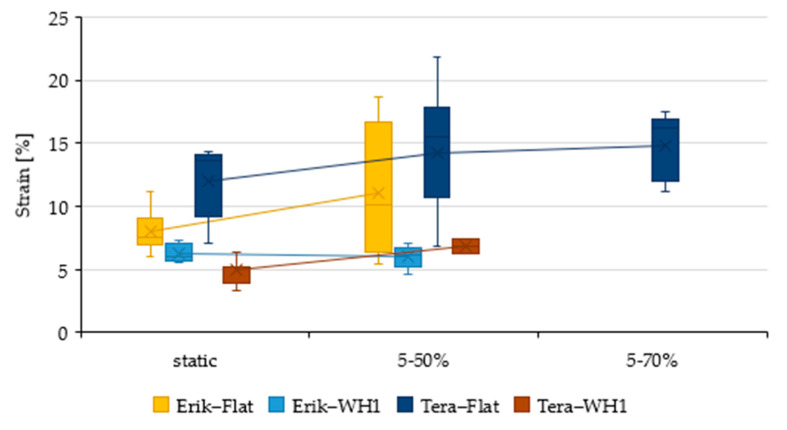
Evaluation of the strain of the adhesive bonds under static loading and cyclic loading in the load intervals 5–50% and 5–70%.

Erik-Flat withstood cyclic loading in the 5–50% interval with a moderate increase in strength to 7.13 ± 0.52 MPa, as shown in Figure 6. The deformation increased to 12.97 ± 4.06% at the same time, as shown in Figure 7. The increased deformation leads to endurance of the bond during cyclic loading. The bond did not reach the parameters high enough to withstand 5–70% load.

Erik-WH1 showed strength (5.29 ± 0.32 MPa) and did not change significantly as compared to static tensile strength, as shown in Figure 6. Deformation also did not show significant changes (5.99 ± 0.80 MPa), as shown in Figure 7. Parameters were sufficient to withstand 5–50% load, but they were not sufficient to withstand 5–70% load. Due to lower strength and higher deformation, the adhesive bonds did not pass the cyclic loading. Both Tera-Flat and Tera-WH1 showed enhanced tensile strength during cyclic loading, along with reduced deformation. This shows a self-reinforcing effect, as shown in Figure 6 and Figure 7.

Tera-Flat exhibited 7.45 ± 0.01 MPa strength and 14.15 ± 2.82% deformation during 5–50% cyclic loading. It was even higher during 5–70% cyclic loading. The strength of 7.49 ± 0.29 MPa together with 14.76 ± 2.41% deformation was observed. Strength of sample Tera-WH1 increased to 5.66 ± 0.40 MPa together with the deformation of 6.79 ± 0.58%. The bond did not withstand 5–70% cyclic loading. Tera-WH2 did not withstand any cyclic loading.

It is evident from Table 6 that the Erik and Tera reinforcements positively influenced the service life and therefore the safety of the adhesive bonds, especially for the bonds marked as Flat and WH1, which correspond with the modulus of elasticity results. Similar results, showing an increase in the service life and safety of the adhesive bonds under cyclic loading by the formation of a composite adhesive layer, have been found by other studies [35,59,60].

**Figure 8:**
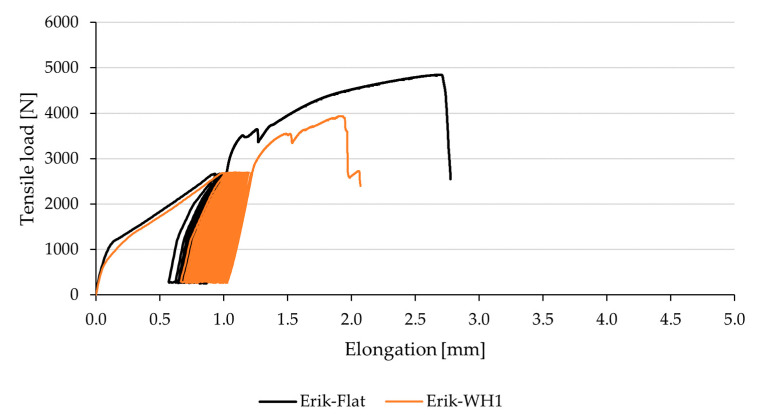
Viscoelastic behavior of adhesive bonds with reinforcing fabric Erik and different shapes during 5–50% cyclic load.

**Figure 9:**
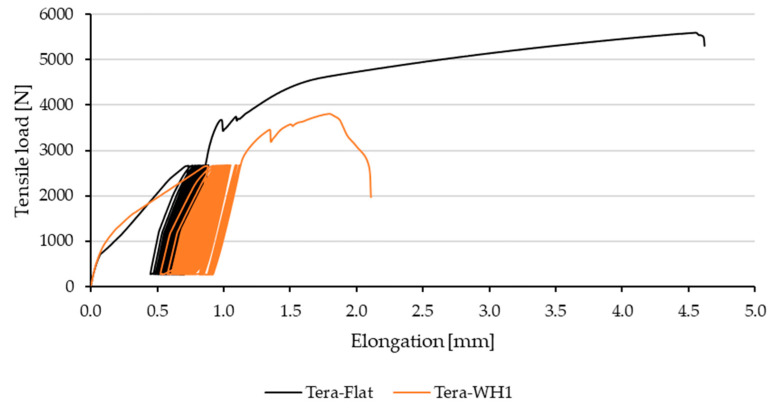
Viscoelastic behavior of adhesive bonds with reinforcing fabric Tera and different shapes during 5–50% cyclic load.

Figure 8 demonstrates viscoelastic behavior (creep) of Erik-Flat and Erik-WH1 during the 5–50% cyclic loading. It clearly shows continuous extension during cyclic loading corresponding to continuous bond fatigue. The longer the extension, the sooner the bond breaks and does not withstand the given number of cycles (1000 cycles). Figure 8 also shows that Erik-WH1 suffered longer extension, which results in lower endurance of the bond. Figure 9 demonstrates cyclic loading of Tera-Flat and Tera-WH1. The behavior is similar to Erik-WH1 (Figure 8). Tera-Flat undergoes lower extension during the cyclic loading, resulting in enhanced capacity for subsequent maximum load. Thanks to this characteristic, Tera-Flat withstood the 5–70% cyclic load.

**Figure 10:**
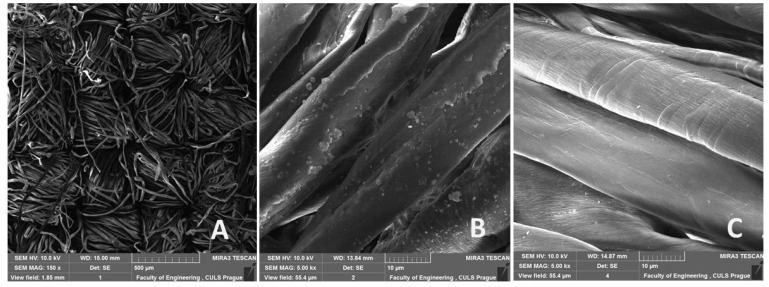
SEM images: (**A**): Cotton fabric Erik (MAG 150×); (**B**): Detailed look at the fabric—warp (cotton fiber) without alkali treatment (MAG 5000×); (**C**): Detailed look at the fabric—warp (cotton fiber) with alkali treatment in 10% NaOH solution for 30 min (MAG 5000×).

Figure 10A shows a microscopic view of the Erik cotton fabric that was used as reinforcement in an adhesive bond. Figure 10B,C show apparent details in microstructures of Erik fabric before and after alkali treatments, respectively. By analyzing the scanning electron microscopy (SEM) images, it was proved that alkali treatment dissolves surface layers of lignin from the cotton fibers in the fabric. The Figure 10C also shows no disintegration of the fiber bundles caused by the NaOH solution treatment. Disintegration of the fibers due to alkali treatment is negative [36,61] and may have a significant negative impact on the mechanical properties of the fibers in the fabric [62,63,64,65].

**Figure 11:**
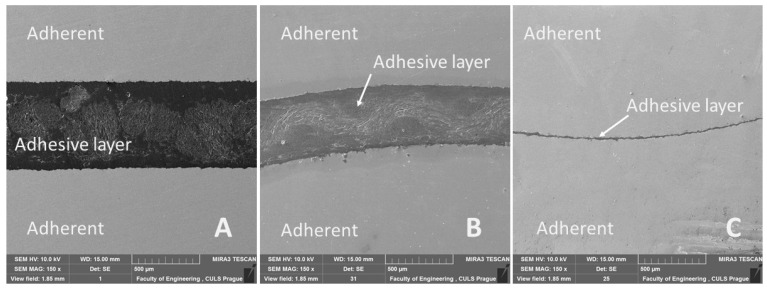
SEM images of samples cut through the adhesive bond: (**A**): cut through Tera-Flat, 0 cycles (MAG 150×), (**B**): cut through Erik-WH1, 1000 cycles in interval 5–50% (267–2674 N) (MAG 150×), (**C**): cut through Resin-WH2, 0 cycles (MAG 150×).

The cross-section of adhesive bonds presented in Figure 11 clearly shows the difference between each tested variant of adhesive bonds. It also shows the arrangement of adherent and adhesive layers in the bonded material. Adhesive layer in Figure 11A,B is composite, consisting of reinforcing cotton fabric Erik/Tera and resin (structural two-component epoxide resin). Furthermore, it demonstrates that every variant of the experiment had a different thickness of the adhesive layer, listed in Table 4. The cross-sections (Figure 11A,C) show integrity of adhesive layer, which was not exposed to the cyclic loading, and Figure 11B shows adhesive layer exposed to 1000 cycles in 5–50% intervals (267–2674 N).

**Figure 12:**
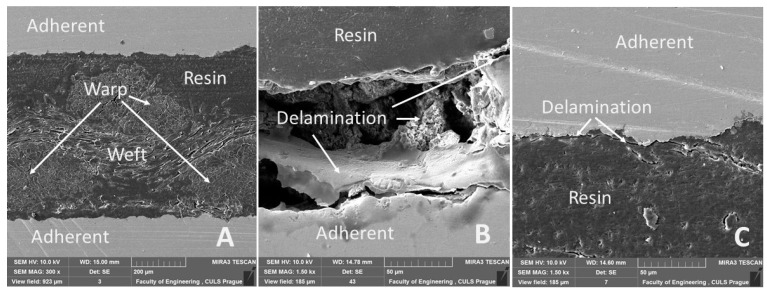
SEM images of cross-section through adhesive bonds: (**A**): cut through Tera-Flat reinforced bond, 1000 cycles in interval 5–70% (267–3743 N) (MAG 300×); (**B**): cross-section of Erik-WH1, 1000 cycles in interval 5–50% (267–2674 N) (MAG 1500×); (**C**): cross-section of Tera-Flat, 1000 cycles in interval 5–70% (267–3743 N) (MAG 1500×).

**Figure 13:**
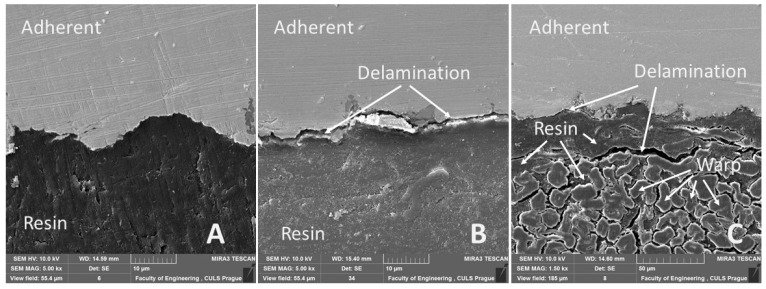
SEM images of cross-section of tested adhesive bond: (**A**): cross-section of Resin-Flat (MAG 5000×), (**B**): cross-section of Erik-WH1, 1000 cycles in interval 5–50% (267–2674 N) (MAG 5000×), (**C**): cross-section of Tera-Flat, 1000 cycles in interval 5–70% (267–3743 N) (MAG 5000×).

From the cross-section of the adhesive bond presented in Figure 12A, the warp and weft of Tera fabric bonded with the resin is visible. It shows the intimate interaction of resin and reinforcing fabric consisting of cotton fibers along warp and weft. Figure 12A–C and Figure 13A show good wettability of bonded material (adherent) with resin. Wettability defines the basic assumption of quality in adhesive bonds [66,67,68]. A detailed look at Figure 12C reveals a slight delamination at the adhesive layer and adherend boundary in identical adhesive bonds due to cyclic loading. Figure 12B shows obvious delamination due to cyclic loading in adhesive layer. Not only is the damage to adhesive layer visible, but it also shows damage to the bonded material. Delamination in any part of an adhesive bond leads to the possibility of rupture and thus damaging the integrity of the adhesive bond, leading to failure [69]. Research results reveal that Tera-Flat after treatment with 10% NaOH solution for 30 min demonstrates improved service life of adhesive bonds through 1000 cycles in intervals 5–50% (267–2674 N) and also in 5–70% (267–3743 N), as shown in Table 5.

Results of the SEM cross-section analysis focused on evaluating adhesive bonds exposed to dynamic loading during cyclic tests. The images demonstrated initiation of micro-cracks in adhesive bond, which leads to delamination. Small cracks appeared inside the adhesive layer (Figure 13C), as well as on the boundary between the adhesive layer and bonded material (Figure 13B). Adhesive bonds that were not exposed to cyclic loading showed no micro-cracks after SEM analysis.

The research involved cyclic tensile testing of wavy-shaped adhesive bonds to understand the fatigue and service behavior. A number of investigations have been carried out regarding adhesive bonds with modified adherend shapes under static loading [26,70]. However, in practical applications, the cyclic loading of non-flat geometries is more relevant. The practical solutions involve several curved elements which undergo cyclic loading and deformation. Sometimes, there is the necessity to create shaped bonds, which reduces the strength substantially. Extensive research needs to be carried out with several other shapes to find practical solutions that suit the design requirements while exhibiting good mechanical performance and service life.

## 4. Conclusions

Experimental results of wavy-lap bonds with natural cotton fabric reinforcement under cyclic loading proved that:

- Wave-shaped bonds WH1 and WH2 reduced the overall strength of the resin under static tests. For Resin-WH1, the strength decreased by 47% to 3.91 ± 0.23 MPa. For Resin-WH2, the strength decreased by 67% to 2.45 ± 0.13 MPa. Resin-Flat, WH1, and WH2 failed the cyclic tests.
- The reinforcing fabric has a positive effect on the mechanical performance of the adhesive bonds. The reinforcing fabrics Erik and Tera did not increase the overall strength of the bond but positively reduced the deformation of the bond and thus increased the elastic modulus and service life of the adhesive bonds under cyclic loading. Erik-Flat and Erik-WH1 passed the 5–50% (267–2674 N) cyclic tests. Tera-Flat and WH1 also passed the 5–50% (267–2674 N) cyclic test. Tera-Flat further passed the 5–70% (267–3743 N) cyclic test.
- SEM analysis showed a positive effect of alkali treatment (10% aqueous NaOH solution) on the fabric surface. The unwanted layers of lignin, oils, and fats were removed. The SEM analysis showed improved wettability of the reinforcing fabrics Erik and Tera due to the alkali treatment with 10% NaOH solution. The SEM analysis also showed the formation of micro-cracks with subsequent delamination due to cyclic loading at the adhesive/adherend interface and at the matrix/reinforcement interface.
- The results of this research demonstrate the ability of natural fabrics to act as reinforcement to increase the service life and safety of hybrid adhesive bonds under cyclic loading. Hybrid adhesive bonds create an interesting alternative in the design of adhesive bonding technology. The use of shaped design for the overlapped bonds is an interesting area that needs to be studied further.

## Figures and Tables

**Table 1:** Basic mechanical properties of the S235J0 steel at 20 °C temperature [47].

Tensile Strength	340–470 MPa
Yield strength	225–235 MPa
Elastic modulus	212 GPa
Elongation	24%

**Table 2:** Indicative chemical composition of S235J0 steel.

C (%)	Mn (%)	P (%)	S (%)	Cu (%)	N (%)	Fe (%)
≤0.19	≤1.50	≤0.04	≤0.04	≤0.60	≤0.014	≤99.55

**Table 3:** Reinforced fabric characteristics [46].

Fabric	Geometry	Areal Density	Warp-Way Strength (200 × 50 mm)	Weft-Way Strength (200 × 50 mm)
		g × m^−2^	N	N
Tera	Plain	290	950	900
Erik	Plain	190	850	800

**Table 4:** Types, shape, and adhesive layer thickness of the hybrid adhesive bonds.

Bond Type	Adherend Geometry (Shape)	Adhesive Layer Thickness (μm)	Characteristics
Resin	Flat	33 ± 3	Adhesive bonds with pure resin and flat shape, WH1 and WH2
	WH1		
	WH2		
Erik	Flat	432 ± 12	Adhesive bonds with composite layer with Erik fabric and flat shape, WH1 and WH2
	WH1		
	WH2		
Tera	Flat	614 ± 9	Adhesive bonds with composite layer with Tera fabric and flat shape, WH1 and WH2
	WH1		
	WH2		

**Table 5:** Results of static tensile tests of adhesive bonds and statistical evaluation of data (*p*-value).

Adhesive Bond		Static Test					
		Tensile Strength		Strain		Modulus of Elasticity	
	Shape	MPa	*p*-value	%	*p*-value	MPa	*p*-value
Resin	Flat	7.38 ± 0.22	-	14.30 ± 1.88	-	52.46 ± 6.65	-
	WH1	3.91 ± 0.23	0.01	4.51 ± 0.73	0.01	88.96 ± 16.02	0.01
	WH2	2.45 ± 0.13	0.01	3.92 ± 0.54	0.01	63.67 ± 8.14	0.01
Erik	Flat	6.53 ± 0.38	0.01	8.00 ± 1.59	0.01	83.68 ± 10.87	0.01
	WH1	5.31 ± 0.29	0.01	6.27 ± 0.65	0.01	85.18 ± 5.29	0.01
	WH2	2.99 ± 0.33	0.01	6.69 ± 3.12	0.01	52.48 ± 17.16	0.50
Tera	Flat	7.12 ± 0.74	0.22	12.03 ± 2.70	0.07	61.53 ± 10.43	0.06
	WH1	4.30 ± 0.83	0.01	4.92 ± 0.91	0.01	87.61 ± 7.91	0.01
	WH2	2.67 ± 0.43	0.01	7.16 ± 3.14	0.01	44.99 ± 19.14	0.19

**Table 6:** Results of cyclic tensile tests of adhesive bonds in the load intervals 5–50% and 5–70%.

Adhesive Bond		Cyclic Test (5–50%)			Cyclic Test (5–70%)		
		Tensile Strength	Strain	Finished Test Samples (1000 cycles)	Tensile Strength	Strain	Finished Test Samples (1000 cycles)
	Shape	MPa	%		MPa	%	
Resin	Flat	-	-	0/7	-	-	0/7
	WH1	-	-	0/7	-	-	0/7
	WH2	-	-	0/7	-	-	0/7
Erik	Flat	7.13 ± 0.52	12.97 ± 4.06	7/7	-	-	3/7
	WH1	5.29 ± 0.32	5.99 ± 0.80	7/7	-	-	0/7
	WH2	-	-	0/7	-	-	0/7
Tera	Flat	7.45 ± 0.01	14.15 ± 2.82	7/7	7.49 ± 0.29	14.76 ± 2.41	7/7
	WH1	5.66 ± 0.40	6.79 ± 0.58	7/7	-	-	0/7
	WH2	-	-	0/7	-	-	0/7

## Data Availability

Not applicable.
